# Recycled Aggregate: A Viable Solution for Sustainable Concrete Production

**DOI:** 10.3390/ma15155276

**Published:** 2022-07-30

**Authors:** Markssuel Marvila, Paulo de Matos, Erich Rodríguez, Sergio Neves Monteiro, Afonso R. G. de Azevedo

**Affiliations:** 1 Advanced Materials Laboratory (LAMAV), UFV—Federal University of Viçosa Campus Rio Paranaíba (UFV-CRP), Rodovia BR 230 KM 7, Rio Paranaíba 38810-000, Brazil; markssuel@hotmail.com; 2 Department of Structures and Civil Construction, UFSM—Federal University of Santa Maria, Coordenadoria Acadêmica, Rodovia Taufik Germano, 3013, Cachoeira do Sul 96503-205, Brazil; paulo.matos@ufsm.br (P.d.M.); erich.rodriguez@ufsm.br (E.R.); 3 Military Engineering Institute, IME—Materials Science Program, Praça Gen. Tibúrcio, 80, Urca, Rio de Janeiro 22290-270, Brazil; snevesmonteiro@gmail.com; 4 LECIV—Civil Engineering Laboratory, UENF—State University of the Northern Rio de Janeiro, Av. Alberto Lamego, 2000, Campos dos Goytacazes 28013-602, Brazil

**Keywords:** construction and demolition waste, recycled aggregate, concrete, sustainable construction

## Abstract

Construction and demolition activities consume large amounts of natural resources, generating 4.5 bi tons of solid waste/year, called construction and demolition waste (C&DW) and other wastes, such as ceramic, polyethylene terephthalate (PET), glass, and slag. Furthermore, around 32 bi tons of natural aggregate (NA) are extracted annually. In this scenario, replacing NA with recycled aggregate (RA) from C&DW and other wastes can mitigate environmental problems. We review the use of RA for concrete production and draw the main challenges and outlook. RA reduces concrete’s fresh and hardened performance compared to NA, but these reductions are often negligible when the replacement levels are kept up to 30%. Furthermore, we point out efficient strategies to mitigate these performance reductions. Efforts must be spent on improving the efficiency of RA processing and the international standardization of RA.

## 1. Introduction

Concrete is the second most used material by volume on Earth. Portland cement is the main binder of concrete, and its production (estimated at 4.3 bi ton/year) accounts for about 8% of the global CO_2_ emission [1]. Besides the CO_2_ emission released by cement production, large amounts of sand and gravel are extracted to serve as aggregates—it is estimated that the natural aggregate (NA) global consumption is of 32 bi tons/year, and this number grows by ~5% per year [2]. The extraction of NA generates environmental liabilities related to exposure of the natural soil layer, erosion processes, deforestation of extraction areas, siltation of rivers, exposure of the groundwater table, among others [3].

In addition to the high environmental impact caused by concrete production, construction and demolition activities generate large amounts of solid waste called construction and demolition waste (C&DW). It is estimated that this kind of waste accounts for 30–40% of the total solid waste generated worldwide, with a global generation of around 4.5 bi ton/year [4,5]. For instance, The European Union, US, and China, respectively, are responsible for the generation of 924 mi, 600 mi, and 2.36 bi tons of C&DW every year [4,5]. Despite the efforts to reuse this waste, about 35% of it is disposed of in landfills [6].

Several other wastes can be cited as problematic from an environmental point of view and are studied for application as recycled aggregates (RA). This is the case with glass waste [7], PET and other plastic waste [8], slag waste [9], and ceramic waste [10]. There are also great efforts to reuse this waste, mainly in the form of aggregates; although some have some binding power, civil construction still does not substantially use recycled waste in the production of concrete and other construction materials.

**Figure 1:**
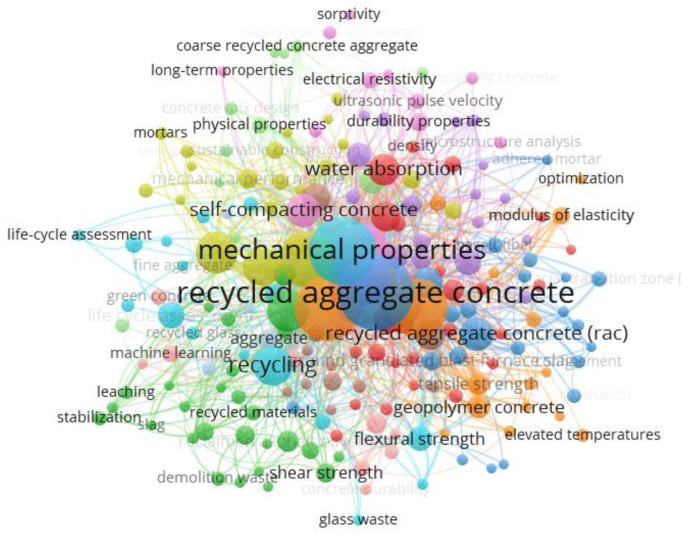
Bibliometrics analysis of concrete containing recycled aggregate for the years 2013–2022.

Figure 1 presents the results of bibliometrics from the analysis of the research carried out on the subject. The keywords present in Figure 1 were obtained using the Scopus database in research between the years 2013 and 2022 and considering the articles published in the main journals in building materials. Input keywords were: recycled aggregate, concrete (or mortar), and sustainable construction. Based on this, 1580 documents were obtained, as indicated in Figure 1.

From the point of view of the construction materials studied, it is observed that the most common are concrete, mortar, geopolymers, and asphalt materials. Regarding the properties studied, the mechanical properties, water absorption, modulus of elasticity, physical properties, electrical resistivity, sorptivity, ultrasonic pulse speed, flexural strength, durability, and equivalent tests stand out. Regarding the materials used, the most outstanding are recycled concrete aggregates (RAC) and construction and demolition waste (or synonyms), followed by glass waste and slag waste.

In the case of glass waste, there are recent studies evaluating the application of the material in cement and geopolymer concrete, such as the study conducted by Siddika et al. [11]. In this study, the authors highlight information on the amount of glass waste generated worldwide and highlight the versatility of the material, which can be used as coarse or fine aggregates or as a binder if the material’s granulometry is appropriate. Another relevant study recently published was conducted by Ferdous et al. [12], where the authors highlight information related to global waste generation, performance, application, and future opportunities on glass waste and other similar materials.

Materials such as ceramic waste and PET waste appear to a lesser extent in Figure 1. Therefore, studies with these materials need to be carried out. One of the reasons to explain the low number of studies on ceramic waste is the fact that the material has pozzolanic potential [13]. However, in the case of PET waste, the low number of studies on the material is not justified, mainly because it is a light aggregate whose use around the world is very considerable.

Through Figure 1 and the highlighted analysis, it is observed that the main gaps in the literature are: evaluating wastes less used in construction materials, such as PET, ceramic, and glass; and verifying relevant properties and parameters that should be studied in other applications of construction materials, in addition to cementitious materials such as concrete and mortar. Particularly noteworthy are the studies on asphalt pavement and geopolymer.

In this context, the use of (RA) from C&DW and other wastes in NA replacement for concrete production has drawn the attention of the technical and scientific communities in the last years. This solution can simultaneously mitigate two major problems: (i) reduce the environmental impact associated with aggregate extraction, and (ii) provide a proper destination for solid waste generated on a large scale worldwide, such as C&DW [7], glass, slag, PET, and ceramic wastes. In this paper, we shortly review the use of RA from C&DW and other wastes for sustainable Portland cement-based concrete production, drawing the main challenges and outlook related to this topic.

The main novelties of the manuscript are: (i) detailing viable solutions to mitigate problems related to the use of recycled aggregates; (ii) presenting alternative solutions to the main problems encountered in RA; (iii) evaluating the potential of other wastes (e.g., ceramic and PET) for application as recycled aggregates. The analysis of these potential wastes was based on a bibliometric study, as highlighted in the previous paragraphs.

## 2. Current Application of C&DW in Construction Materials

### 2.1. Application in Concrete

C&DW can be used in concrete as coarse/fine aggregate and a finely ground material (i.e., filler). Concerning aggregate, Robalo et al. [14] observed that replacing NA with C&DW progressively increases the water or superplasticizer admixture to keep the workability of concrete due to the high porosity and water absorption of C&DW. However, Rashid et al. [15] found that pre-saturating the C&DW avoided this workability loss. This pre-saturation can also improve the resistance to internal steel corrosion [16]. Duan et al. [17] replaced 0–100% NA with coarse C&DW in self-compacting concrete, observing that 25% replacement did not change the compressive strengths, while the flexural strength decreased as the aggregate replacement level increased. In turn, Sahoo and Singh [18] evaluated the punching shear capacity of C&DW concrete slab-column connections, observing that the punching-shear crack angle and the punching strength were independent of the C&DW replacement level for a given strength class. As for durability, Duan et al. [17] found that no significant damage in the concrete resistance to chloride penetration was observed for up to 50% NA replacement with C&DW. Similarly, Cantero et al. [19] observed equivalent electrical resistivity (associated with the resistance against chlorite ions migration) for concretes containing 100% NA and 50% NA + 50% C&DW. Sáez del Bosque et al. [20] evaluated the carbonation depth of concretes with 0–50% replacement of NA with coarse C&DW, finding statistically equivalent values for the control (100% NA) and 25% C&DW-containing concretes.

**Figure 2:**
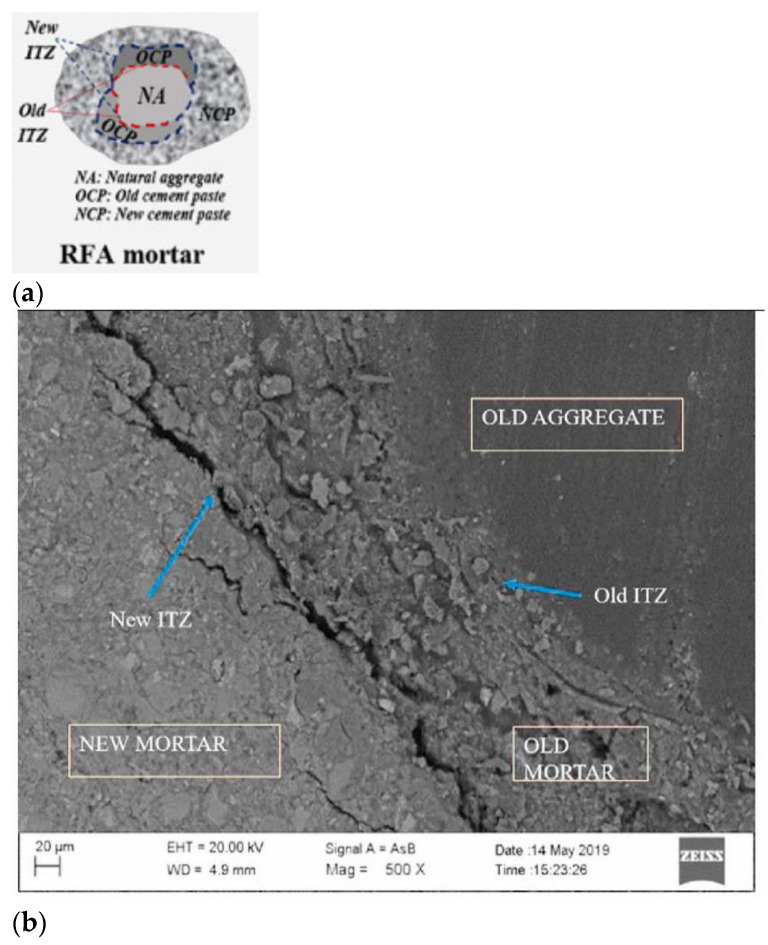
Schematic illustration of C&DW in concrete [22] in (**a**); scanning electron microscopy of mortar containing 40% C&DW [23] in (**b**).

Overall, we can observe that the fresh and mechanical properties of concrete are negatively affected by high replacement levels of NA with C&DW, while durability aspects are not so sensitive to it [21]. The worse performance of C&DW can be associated with its surface characteristics, which usually contain old mortar (harming the interfacial transition zone–ITZ–with the new cementitious matrix), in addition to microcracks generated during C&DW crushing [4]. These aspects are illustrated in Figure 2. However, concrete properties are marginally affected when the replacement levels are kept relatively low (e.g., up to 30%).

**Figure 3:**
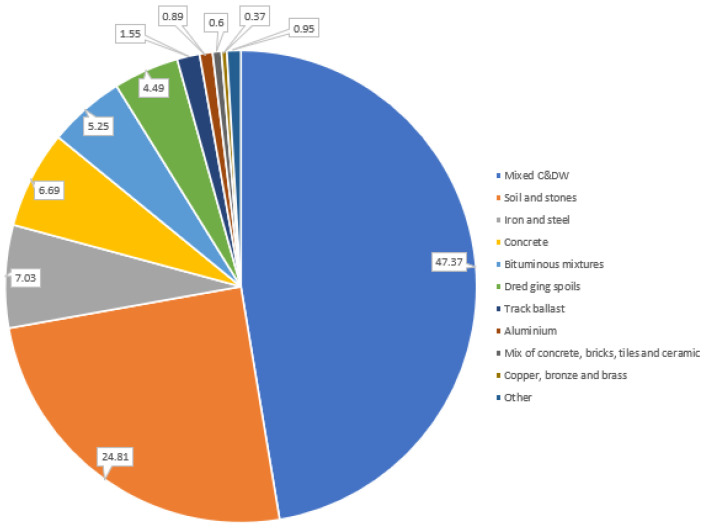
C&DW composition produced in Naples, Italy.

Another negative feature of C&DW is its heterogeneity [24]. Exemplifying this negative aspect, Figure 3 presents the main fractions composing the amount of C&DW obtained in the city of Naples in Italy [25]. It is observed that the C&DW has 47.37% mixed material, 24.81% soil and stones, 7.03% iron and steel, 6.69% concrete, and 5.25% bituminous mixers, among other minor components. These material grades vary by C&DW, but it is important to understand through Figure 3 that the material is very heterogeneous. This aspect represents yet another difficulty in the use of this type of RA. Thus, it is highlighted that, in addition to trying to solve the problem related to adhering grout to the aggregate grains highlighted in Figure 2, alternatives must be considered to solve the problems related to the heterogeneity of C&DW.

Alternatively, C&DW can be finely ground to a “micro-aggregate”, i.e., filler. In fact, recent studies [25] confirmed that carbonated cement paste presents amorphous aluminosilicate gel with pozzolanic properties. De Matos et al. [26] observed that powder C&DW led to 5% higher compressive strengths than limestone filler for the same incorporation level, and that the residue acted mainly as an inert filler within the first seven days of hydration. This was confirmed later by Frías et al. [27], who observed that after 28 days, extra C-S-H and ettringite were formed in the presence of powder C&DW, indicating the potential binding activity of the material [28,29]. This potentially improves the ITZ between RA and the new cementitious matrix [30], compensating for the negative effect on ITZ mentioned earlier. Cantero et al. [19] simultaneously replaced 10–25% Portland cement with ground recycled concrete (GRC) and 0–50% NA with C&DW RA, evaluating the mechanical behavior of concrete. The authors found that the concrete containing 10% GRC and 50% C&DW RA achieved the same strength class as the 100% Portland cement concrete. He et al. [31] observed that replacing 30% binder with GRC reduced the autogenous shrinkage of ultra-high-performance concrete while leading to comparable compressive strengths (123–128 MPa). In general, we can observe that the use of C&DW powder as filler tends to keep or even improve the properties of concrete.

### 2.2. Application in Other Construction Materials

In addition to the application of C&DW as RA in concrete, highlighted in topic 2.1, these materials can be applied to other building materials. These other applications will be highlighted in this section. Table 1 presents some works that evaluated the use of C&DW as RA in different construction materials, except concrete and mortar. Table 1 was developed considering the database evaluated in the bibliometric study highlighted in Figure 1 and prioritizing publications carried out in more recent years.

From the information presented in Table 1, it is possible to observe that the main applications of C&DW as RA in materials other than concrete are asphalt pavement, pavement subbase, or geopolymers. It is worth noting that in this type of application, the concerns regarding the controlled properties are different. While in concrete, the main properties are related to mechanical behavior, in the case of the applications illustrated in Table 1, it is important that parameters such as water absorption, frost resistance, friction, and hardness are considered. As a result, replacement levels of C&DW and RA can reach up to 100%.

In the case of asphalt pavement application, in general, C&DW is used as coarse aggregate. Although it is not the most relevant property, it is important that there is an analysis of the mechanical properties of asphalt pavement with C&DW as RA, as highlighted by Zhu et al. [41]. Another relevant issue is the transition zone between the RA and the asphalt pavement. This transition zone, studied by Hu et al. [32], also presents adhesion problems through the same mechanisms highlighted in Figure 2. Therefore, another aspect that must be carefully studied is the morphology of the RA because it directly affects the adhesion with asphalt pavement. This was the point of the investigation by Guo et al. [39], who verified the direct influence between the morphology of the RA and the mechanical behavior of the asphalt pavement due to the deficiencies of the IZT. A positive point highlighted by Guo et al. [39] is that the RA, in general, is irregular and, therefore, has good adhesion with asphalt pavement.

**Figure 4:**
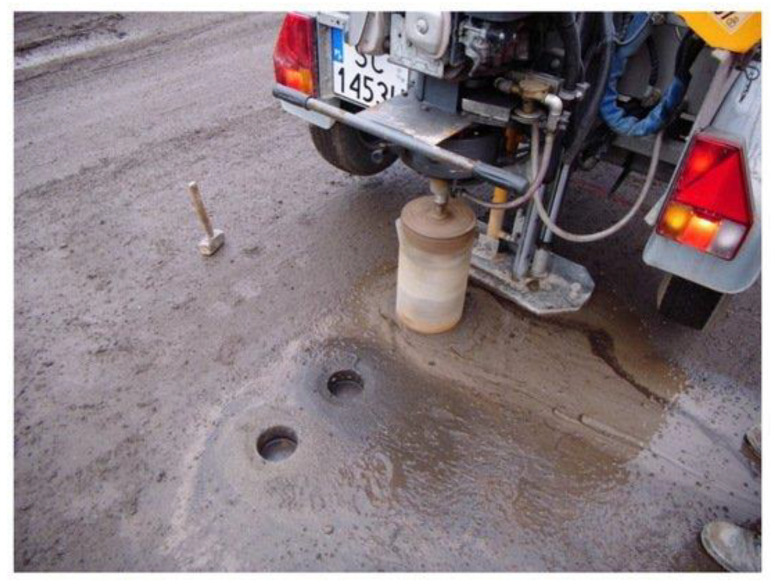
Pull-out test on asphalt pavement with RA.

Two important properties that should be studied when applied to asphalt pavement are resistance to high temperatures and adhesion with the pavement. Hu et al. [33] emphasize that the investigation of the stability at high temperatures of asphalt mixture with RA should be considered to enable the application of this material. Without this property being proven, it is impossible to use RA in this application, since asphalt pavements are commonly mixed at high temperatures during their application. Regarding adherence, Slabonski et al. [40] highlight that this evaluation must be carried out through a pull-out test, as illustrated in Figure 4. As the asphalt pavement is often subjected to intense traffic loads, the material’s natural tendency is to lose adhesion. Therefore, this is another property that differentiates the study of the application of C&DW in asphalt pavements and in concrete.

Another possible application is in pavement subbase. In this case, a property of great relevance that needs to be controlled is the resilient modulus. Corradini et al. [43] evaluated the application of C&DW as aggregate coarse in subbase pavement, studying the differences found in the resilient modulus. The authors observed that the use of C&DW is viable in this type of application since the values obtained from the resilient modulus are compatible with the application in pavement subbase. These results are consistent with the research carried out by Tefa et al. [42]. In the authors’ study, it is highlighted that, although there is still skepticism among designers, contractors, and road agencies, the application of C&DW as an aggregate in subbase pavement should not be neglected because the material properties are compatible with this application and due to the high environmental gain provided by this application.

Another highly researched application for C&DW as RA is geopolymers. It should be noted that these materials are defined as structural materials used in the same applications as OPC concrete [50]. However, geopolymers are obtained through the geopolymerization reaction between a precursor, usually rich in aluminosilicates, and an alkaline activator solution [51,52]. In the study of these materials, it is necessary to use fine aggregate and coarse aggregate, and C&DW is viable for this function.

It is noteworthy that in the application of C&DW in geopolymers, the same properties evaluated in concrete must be considered; that is, the mechanical properties of the material must be evaluated. Saba and Assaad [45], for example, evaluated the effect of using recycled fine aggregates in geopolymers at levels of up to 60%. The authors concluded that the results obtained are compatible with the application proposed in the research, in mortar for masonry, and verified that the use of RA helps in the retention of water in the material, which is a beneficial factor due to the high content absorption caused by masonry. Positive results were also obtained in the studies by Rahman et al. [46] and Xie et al. [47], where the authors stated the possibility of using C&DW as RA in geopolymers.

**Figure 5:**
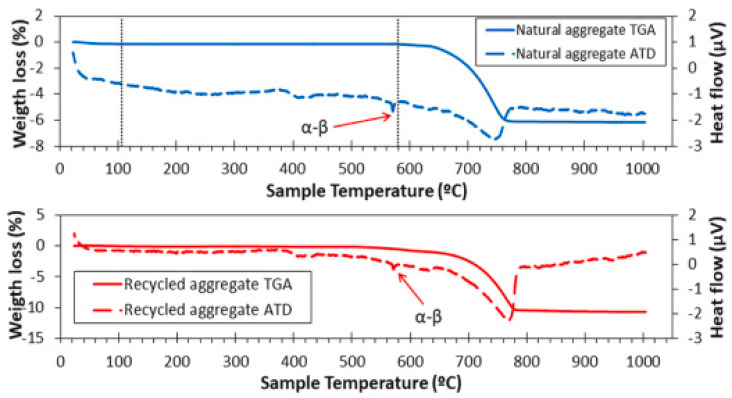
Weight loss of NA and RA.

However, some negative results were also reported by authors in the application of C&DW to RA in geopolymers. In general, these materials positively resist the effects of high temperature. However, with the use of C&DW as AR, this effect is harmful, as highlighted by Pawluczuk et al. [49]. The authors evaluated the mass loss of an NA and an RA based on C&DW, as highlighted in Figure 5. However, while NA had a maximum mass loss of approximately 6%, the RA had a loss of almost 10%. This is due, in part, to the transformation of quartz from a to b at around 600 °C. Therefore, it is observed that the use of C&DW as RA in geopolymers subjected to high temperatures is not indicated.

## 3. Environmental Benefits of Using RA from C&DW

The environmental benefits of using RA rely on three main aspects. First, it allows for reducing the natural resource demand and the CO_2_ emission associated with aggregate extraction and processing, in addition to the impacts related to cement production when C&DW is used as a ground powder in cement replacement. For example, Cantero et al. [19] evaluated the CO_2_ emission of the mixes containing GRC and C&DW RA mentioned earlier, observing emission reductions of up to 19.7% compared to plain Portland cement + NA concrete. The second aspect is related to the CO_2_ “capture” or “sequestration” by C&DW [53]. Portland cement reaction produces Ca(OH)_2_ as one of the hydration products, which is converted into CaCO_3_ in contact with the CO_2_ from the atmosphere, known as carbonation [28]. Therefore, cement-based elements (e.g., rendering mortars and concrete elements) capture CO_2_ from the atmosphere and, using C&DW RA, trap it inside new concrete. Finally, considering the amount of C&DW generated worldwide, and that ~35% is disposed of in landfills, we can estimate that around 1.5 bi tons of C&DW are disposed of every year. Even a partial replacement of NA with RA in concrete would help to give a proper destination for this residue generated on a large scale, in addition to increasing its added value.

## 4. Challenges and Outlook

There are some challenges in effectively using RA in concrete. The first to be mentioned is the mechanical strength reduction of concrete discussed earlier. To overcome these strength reductions, there are currently three main strategies:

(i) Reducing the content of old mortar in RA: Saravanakumar et al. [54] pre-treated RA with sulfuric, nitric, and hydrochloric acids, achieving better mechanical performance in concrete than untreated RA. Thermal treatment was also used for this purpose [55], but high energy intake may be required to reach the desired decomposition temperatures (around 800 °C).
(ii) Surface-treating RA before its application in concrete: He et al. [56] found that the density, water absorption, and crushing index of RA were improved with previous treatment using pozzolan slurry combined with sodium silicate and silicon-based additives, improving its performance in concrete. Li et al. [57] pre-treated RA with nanosilica suspension (spraying and soaking), observing that the micro-hardness of both the old mortar and the new mortar near the ITZ was enhanced after treatment, improving the compressive strength, water absorption, and chloride penetration resistance of RA-containing concrete. Zhang et al. [58,59] observed that pre-treating RA with a sulfoaluminate cement slurry led to a denser RA surface (with higher micro-hardness), leading to improved mechanical strength and durability.
(iii) Compensating concrete performance loss using fibers: Zong et al. [60] observed that 1.2% steel fiber incorporation evened or increased the 28-day flexural strength of concretes containing 50–100% RA compared to 100% NA concrete without fibers. Similarly, Paluri et al. [61] observed 28-day compressive and flexural strength reductions of up to 23 and 18%, respectively, when NA was replaced with 50–100% RA. However, when adding 1% steel fiber, the 50% RA concrete had comparable (5% lower) compressive strength and 31% higher flexural strength than 100% NA concrete. It is worth mentioning that synthetic fibers can be advantageous from a technological point of view; however, in countries with an abundance of natural fibers like Brazil and India, these can be a better solution for the destination of agro-industrial waste [62].

Another central point is the heterogeneity of RA. C&DW can contain several different materials. Souza et al. [40] observed that C&DW was composed of mortar residues (20%), wood (19%), concrete (14%), soil (14%), brick (11%), and steel (9%), besides minor amounts of natural rocks, paper, thermoacoustic tiles, fiber cement sheets, glass, expanded polystyrene, and cement boards. However, the authors demonstrated that this composition varies depending on the construction step (new construction or renovation) as well as the execution phase (structure, envelope, or finishes). Furthermore, Galderisi et al. [63] observed that the petrography of C&DW depends on the geographical area where the waste is produced. In limestone-abundant areas, calcareous materials are found in high contents, while the residue is rich in silica and alumina in limestone-poor areas. In this context, it is hard to predict the mechanical properties of RA and, therefore, its performance in concrete.

One strategy to reduce RA heterogeneity is the pre-separation of the waste fractions during the different stages of construction, i.e., during the generation of C&DW. Another approach involves RA processing, removing the contaminants listed above and preferentially the adherent mortar attached to it, besides crushing it to an adequate particle size distribution. For this purpose, various methods are available, such as mechanical (jaw, hammer, rolling, impact, and hand crushers), chemical (acid dissolution), and thermal approaches (freeze-thaw, thermal expansion, heating and rubbing, and microwave heating) [64]. While the mechanical methods facilitate large-scale processing, part of the adherent mortar may only be entirely removed by chemical/thermal methods, which are often complex and more expensive to perform at a large scale.

Furthermore, the shortage of international standards addressing RA requirements also impairs their routine use in structural concrete. While EN 206 [65], Annex E, brings specific recommendations for RA properties, many standards, such as Brazilian standard NBR 7211–Aggregates for Concrete: Specification [66], does not even mention RA. This lack of technical support reduces the safety of materials and structures designers to use RA concrete. There is still much misinformation from consumers who associate RA with low-quality construction, which is not true. The stimulus to research and its dissemination become important tools for the use and commercial acceptance of RA.

## 5. RA from Other Wastes

In this section, other wastes with the potential to be used as RA in concrete and other construction materials will be discussed. Table 2 presents a summary of works with these materials, extracted from the same database of the bibliometric analysis of Figure 1. The most recent publications were prioritized to build Table 2.

### 5.1. Glass Waste as RA

Glass Waste is one of the most generated wastes by human beings due to the versatility of glass used in the packaging industry, home appliances, and civil construction [83,84]. Annually, around 14 million tons of waste glass are produced in the European Union and around 11.38 million in the USA [51]. These numbers highlight the urgency of recycling the material.

An important advantage of using glass as RA is the fact that it is easily ground. Compared to other RA such as C&DW, the energy required to reduce the glass waste particle size is much less [68]. However, this can also be a disadvantage in using this material as RA as it is more likely to wear through abrasion [85]. Another interesting advantage of glass waste is that it is more uniform than other ARs within the same production standard. In other words, glass from the packaging industry is homogeneous with each other [68], which is an important advantage for using this material as RA [73].

Another important point of using waste glass as RA is that the material can increase the durability of cementitious materials. Alducin-Ochoa et al. [72] evaluated the durability of mortars produced with glass waste as RA partially replaced NA in contents of up to 25%. The authors observed that glass waste mortars showed superior performance in salt crystallization, freezing, and thermal shock tests. This is another advantage of using the material as AR.

Another important issue made possible using glass waste as RA is highlighted by Xiao et al. [67]. The authors verify the possibility of using glass waste as a luminescent material in decorative mortars. This effect is only possible with the use of glass waste which, if correctly used as an AR, allows the emission of visible light (glow) due to its luminescent surface. Furthermore, in the same study, the authors proved that glass waste has a high mechanical performance when used as an RA.

A worrying disadvantage of using glass waste as RA is the possibility of alkali–aggregate reactions due to the high content of amorphous and reactive silica in the composition of this material. This causes cracks and failures in concrete structures. However, as highlighted by Duan et al. [69], the use of organic waste such as drinking water treatment sludge as a substitute for OPC can reduce this effect. Another possibility to reduce this problem is to promote the curing of cementitious material with glass waste in CO_2_ curing, as highlighted by Whang et al. [71]. This type of curing causes an increase in mechanical strength, a reduction in pH, and permeability of the material. This is because the treatment promotes a reduction in the CH content of the OPC. In the case of glass waste, the treatment promotes the occurrence of an alkali–aggregate reaction on the surface of the glass, generating pressure into the glass aggregate. This pressure acts in the opposite direction of the typical expansion of the alkali-aggregate reaction, mitigating the problem. Therefore, this problem should not be enough to rule out the use of glass waste as AR.

### 5.2. Slag Waste as RA

Slag wastes are materials from different metallurgical processes, such as in producing steel. These materials are known as granulated blast furnace slag and can undergo two types of cooling: abrupt cooling, which allows the material to obtain agglomerating characteristics, thus being a viable substitute for OPC; or slow cooling, where the material loses its binding properties and can only be used as RA [52,86]. Since the binding materials have greater added value, the metallurgical industries started to produce slag waste through an abrupt cooling process, aiming to use it as a binding material.

However, there are still studies that use slag wastes from steel production. For example, the work by Goli [73] is cited, in which the authors used 75% steel slag to produce asphalt pavements. The authors concluded that the use of steel slag improved the marshall stability and tensile strength of the material. Therefore, the use of this material is viable as an AR, even if this is not the best waste application. Another interesting work to mention is that of Chandru and Karthikeyan [74], where the authors used recycled steel slag as coarse aggregate to produce self-compacting concrete. The authors observed that the material presented good mechanical results and good durability parameters.

However, in addition to the granulated blast furnace slag, there are other types of waste that can be used as RA. This is the case with ferronickel slags, which do not have high binding power, as is the case with granulated blast-furnace slag. In this sense, the best application for the material is RA. This was studied by Luo et al. [75] and Petrounias et al. [76], where the authors proved the use of this material in the proposed application.

### 5.3. Ceramic Waste as RA

**Figure 6:**
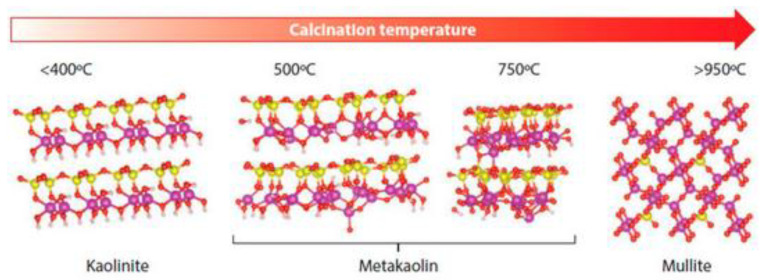
Transformation of kaolinite as a function of calcination temperature.

Ceramic waste is a material obtained by grinding blocks and bricks obtained from construction and demolition, or from the ceramic industry [77]. Depending on the burning range of the blocks in the manufacturing stage, the material may have a crystalline or amorphous structure. This happens due to the transformation of clay minerals, mainly kaolinite, as shown in Figure 6 [87]. This clay mineral can transform into metakaolin, if the temperature occurs in the range of 500–750 °C, which has an amorphous structure. In this case, the material has pozzolanic potential and can be used as a substitute for OPC or as a precursor in geopolymers [10]. If the firing temperature exceeds 950 °C, the material is transformed into mullite, which has a crystalline structure. In this case, it is possible to use the material as RA [87].

Liu et al. [77] evaluated the use of ceramic waste as fillers in the production of concrete. The authors observed that the energy consumption to perform the grinding of the material is very high and should be considered a major disadvantage in the use of the material as RA. However, the results of mechanical properties obtained are very relevant and enable the use of ceramic waste as RA.

In addition to the research by Liu et al. [77], other authors also verified the feasibility of applying ceramic waste to RA. Yang et al. [78] evaluated the properties of foam concrete containing ceramic waste as RA; Aldemir et al. [79] evaluated the shear behavior of concrete beams containing ceramic waste as RA. In both surveys, the results obtained were very satisfactory.

### 5.4. PET Waste as RA

PET waste is a material with great potential for use in AR. However, one of the great difficulties is to perform the grinding of the material due to the surface area of the waste. Furthermore, the energy used for grinding the material is higher than other wastes. This is a major difficulty in using the material [88].

Another problem is the large absorption of water promoted by PET waste. This makes it difficult to use the material at high levels. Campanhão et al. [8] and Silva et al. [80] evaluated the use of PET waste as RA replacing NA in cementitious materials. The authors evaluated the substitution of up to 30% and observed that the use of the residue causes a reduction in the workability and a decrease in the mechanical strength of the material. However, at contents of up to 10% of waste, the authors observed an increase in compressive strength due to an increase in the packing of the material.

Other authors have confirmed this pattern. For example, Perera et al. [81] and Alfahdawi et al. [82] evaluated the use of PET waste at levels of 5 and 2.5%, respectively. Therefore, this pattern of incorporation at low levels is typical of the material. In view of this, it is observed that the main difficulty in using the material is the high-water absorption of the PET waste. This pattern reduces the amount of work done with the material.

### 5.5. Other Waste as RA

Other wastes can also be used as RA. An example is the primary sludge waste from the paper industry. This waste is an organic material that can have a binding potential. As an aggregate, it should be used in low levels due to the high leaching potential [89]. The ornamental stone waste is also mentioned. These materials are generally inert and used as a filler fraction. The main limitation of the material is the high-water absorption, which can impair the mechanical performance of the material. However, it can promote greater mechanical strength due to promoting packing [90,91].

Another waste that can be used as AR are aggregates obtained from geopolymers. As with C&DW, aggregates obtained from geopolymers are a viable option for the development of AR replacing NA. This information was highlighted in the research by Xu et al. [92], where the authors evaluated the possibility of producing artificial aggregates using geopolymer aggregate concrete for light coarse aggregate. Although the mechanical performance obtained with the use of geopolymer aggregate was lower than that of natural aggregates, the scarcity of this material makes the use of this RA class a viable alternative.

These results are consistent with other studies, such as the works by Xu et al. [93] and Qian et al. [94]. In both works, the authors evaluated the use of geopolymer fine aggregates to produce geopolymer materials. However, in these studies, the authors found that the geopolymer fine aggregates showed reactivity with the cement matrix. This resulted in strong interfacial bonding, which is a promising result. It is noteworthy that even though, due to the complexity of the interaction between geopolymer fine aggregates and the geopolymer matrix, the ITZ obtained is not clearly understood. This highlights the need to develop new research in the area to enable the application of geopolymer aggregates to RA.

## 6. Conclusions

Construction and demolition activities use large amounts of natural resources and account for a considerable portion of global CO_2_ emissions. In addition to that, they generate about 4.5 bi tons of solid waste (i.e., C&DW) every year, from which around 35% is disposed of in landfills. The use of RA from C&DW for concrete production can simultaneously mitigate these environmental problems.

It is important to conclude that the article benefits other researchers in the field of recycled aggregates because it presents some solutions to mitigate the main problems related to the use of recycled aggregates. For example, the current literature shows that RA tends to reduce concrete’s fresh and hardened performance compared to NA. However, these performance reductions are often negligible when the replacement levels are kept low, e.g., up to 30%. Durability-related properties are not much affected by RA incorporation. There are some efficient strategies available to mitigate these performance reductions, mainly: (i) reducing the content of old mortar in RA; (ii) treating the RA’s surface before its application in concrete; and (iii) compensating the performance loss through fiber addition. Non-structural concrete is another good candidate for RA application, even at 100% NA replacement.

Owing to the feasibility of using RA for sustainable concrete production, efforts must be spent on improving the efficiency of RA processing, thus allowing large-scale production with lower heterogeneity, in addition to international standardization for this type of aggregate. In addition, this literature review highlighted some pertinent information that allows understanding the behavior of C&DW and the main properties that need to be evaluated in other applications of building materials. Using C&DW as RA in asphalt pavement, pavement subbase, and geopolymer requires greater control of durability properties but allows for the use of 100% RA as coarse and fine aggregate.

Finally, other wastes that can be used as RA were evaluated. This is the case for glass, PET, slag, ceramic, and artificial aggregate. Some of these materials face resistance in the use of AR, as is the case with PET and ceramic waste. As a result, there is a need for further research evaluating the behavior of these materials as recycled aggregates.

## Figures and Tables

**Table 1:** Application of C&DW as RA in building materials.

Construction Materials	Reference	Aggregate Type	Max % Used	Authors
Asphalt pavement	[32]	Coarse	100	Hu et al.
Asphalt pavement	[33]	Coarse	100	Hu et al.
Asphalt pavement	[34]	Coarse	100	Kar et al.
Asphalt pavement	[35]	Coarse	50	Yang et al.
Asphalt pavement	[36]	Coarse and Fine	50	Xu et al.
Asphalt pavement	[37]	Coarse	100	Bittencourt et al.
Asphalt pavement	[38]	Fine	100	Adesina and Das
Asphalt pavement	[39]	Coarse	100	Guo et al.
Asphalt pavement	[40]	Fine and Coarse	100	Slabonsi et al.
Asphalt pavement	[41]	Coarse	100	Zhu et al.
Pavement subbase	[42]	Coarse	100	Tefa et al.
Pavement subbase	[43]	Coarse	100	Corradini et al.
Geopolymer	[44]	Coarse	30	Haoi-Bao et al.
Geopolymer	[45]	Fine	60	Saba and Assaad
Geopolymer	[46]	Fine	100	Rahman et al.
Geopolymer	[47]	Coarse	100	Xiet et al.
Geopolymer	[48]	Coarse	100	Was et al.
Geopolymer	[49]	Coarse	100	Pawluczuk et al.

**Table 2:** Wastes used as RA in construction materials.

Waste	Reference	Aggregate Type	Max % Used	Authors
Glass	[67]	Fine and Coarse	100	Xiao et al.
Glass	[68]	Coarse	100	Sharma et al.
Glass	[69]	Coarse	100	Duan et al.
Glass	[70]	Fine	30	Zhan et al.
Glass	[71]	Fine	100	Wang et al.
Glass	[72]	Fine	25	Alducin-Ochoa et al.
Slag	[73]	Coarse	75	Goli
Slag	[74]	Coarse	60	Chandru and Karthikeyan
Slag	[75]	Fine	100	Luo et al.
Slag	[76]	Fine	54	Petrounias et al.
Ceramic	[77]	Filler	30	Liu et al.
Ceramic	[78]	Coarse and Fine	30	Yang et al.
Ceramic	[79]	Coarse and Fine	100	Aldemir et al.
PET	[8]	Fine	30	Campanhão et al.
PET	[80]	Fine	30	Silva et al.
PET	[81]	Fine	5	Perera et al.
PET	[82]	Fine	2.5	Alfahdawi et al.

## Data Availability

Not applicable.
